# A Systematic Review of Predictors of Vaping Cessation Among Young People

**DOI:** 10.1093/ntr/ntae181

**Published:** 2024-07-20

**Authors:** Anasua Kundu, Siddharth Seth, Daniel Felsky, Theo J Moraes, Peter Selby, Michael Chaiton

**Affiliations:** Institute of Medical Science, University of Toronto, Toronto, Ontario, Canada; Centre for Addiction and Mental Health, Toronto, Ontario, Canada; Health Science, Queen’s University, Kingston, Ontario, Canada; Institute of Medical Science, University of Toronto, Toronto, Ontario, Canada; Centre for Addiction and Mental Health, Toronto, Ontario, Canada; Dalla Lana School of Public Health, University of Toronto, Toronto, Ontario, Canada; Institute of Medical Science, University of Toronto, Toronto, Ontario, Canada; Hospital for Sick Children (SickKids), Toronto, Ontario, Canada; Department of Paediatrics, University of Toronto, Toronto, Ontario, Canada; Institute of Medical Science, University of Toronto, Toronto, Ontario, Canada; Centre for Addiction and Mental Health, Toronto, Ontario, Canada; Dalla Lana School of Public Health, University of Toronto, Toronto, Ontario, Canada; Department of Family and Community Medicine, University of Toronto, Toronto, Ontario, Canada; Institute of Medical Science, University of Toronto, Toronto, Ontario, Canada; Centre for Addiction and Mental Health, Toronto, Ontario, Canada; Dalla Lana School of Public Health, University of Toronto, Toronto, Ontario, Canada

## Abstract

**Background and Aims:**

Understanding the factors influencing vaping cessation among young people is crucial for targeted interventions. This review aimed to summarize the individual and environmental factors that predict vaping cessation-related behaviors in the young population.

**Methods:**

We systematically searched five databases for studies investigating predictors of vaping cessation behaviors among young people aged 10–35 years. Studies that examined predictors of cessation of cigarettes, other tobacco products, cannabis vaping, and studies evaluating efficacy of cessation interventions were excluded. Quality in Prognosis Studies tool was used to assess risk of bias.

**Results:**

We found 24 studies analyzing predictors of intention to quit vaping (*n* = 15), quit attempts (*n* = 11), and vaping abstinence (*n* = 7). Most studies had low risk of bias, except for study attrition. We identified 107 predictors and grouped them into “probable,” “possible,” “insufficient evidence,” “probably unrelated,” and “inconsistent direction” categories. For “probable” predictors, we found 11 for intention to quit, eight for quit attempts and five for vaping abstinence. Overall, harm perception of vaping, current other tobacco products use, frequency of use, and level of nicotine dependence were common “probable” predictors across three outcomes, with low harm perception of vaping, dual use, and poly tobacco use associated with decreased intention to quit and quit attempts in younger population (~10–19 years).

**Conclusions:**

Predictive modeling studies investigating vaping cessation-related behaviors among young people are still limited. Future research should specifically study the natural history of vaping in youth in different jurisdictions, populations, and age groups to expand our knowledge in this area.

**Implications:**

We identified and categorized predictors of intention to quit vaping, quit attempts, and vaping abstinence among young people. While the “probable” predictors can inform public health and policymakers to plan targeted vaping cessation programs for high-risk populations, raising public harm perception of vaping and encouraging to quit other tobacco products might increase intention to quit and quit attempts among younger population. However, the “possible,” “insufficient evidence” and “inconsistent direction” predictors need further testing by future prospective longitudinal research. Additionally, we emphasized the significance of appropriate study designs, conducting research across various jurisdictions, and different population groups to obtain comprehensive insights.

## Introduction

Globally, the prevalence of electronic cigarette (e-cigarette) use among young people has climbed significantly over the last few years. The rate of lifetime e-cigarette use among people aged 15–19 years and 25–34 years doubled between 2013 and 2020.^1^ The Canadian Tobacco and Nicotine Survey, 2021 reported that 13% of the youths and 17% of the young adults vaped in the past 30 days compared to only 4% of the adults aged 25 or older.^2^ This rise in usage is alarming, particularly because of the new and emerging evidence of the harms of e-cigarettes. In addition to causing nicotine dependence, e-cigarettes have been consistently associated with exposure to several toxic and carcinogenic chemicals, and adverse health effects such as increased respiratory symptoms, increased blood pressure, and increased heart rate.^3–7^ Moreover, regular e-cigarette use was found to increase the risk of subsequent initiation of combustible cigarette use among nonsmokers, further exposing them to harmful toxic chemicals.^3,4,8^ However, these health concerns and additional factors such as costs and concerns about the threat of COVID-19 related harms were found to motivate youth to quit vaping.^9–12^ In 2020, 37% of Canadians aged 15 or older who vaped made attempts to quit vaping in the past year.^13^

While there is controversy on whether vaping cessation should be encouraged among former smokers, several respected health organizations (eg, World Health Organization [WHO], American Lung association, Truth Initiative, Smokefree Teen) have recommended vaping cessation for young people.^4,14–17^ However, little is known about the factors that influence young peoples’ decision to quit and make them successful while quitting. Although a recent systematic review summarized the predictors of vaping cessation,^18^ the review only included experimental and longitudinal studies and did not focus on the young population. Hence, we conducted this review to systematically summarize the individual and environmental factors predicting vaping cessation related behaviors in the young population.

## Methods

We followed the Preferred Reporting Items for Systematic Reviews and Meta-Analyses (PRISMA) guideline adhering to the four-phase flow diagram and the 27-item checklist for this study.^19^ This protocol was registered on the Open Science Framework (https://osf.io/p96mw/).^20^ The review was conducted in five steps: (1) database search and exclusion of duplicate articles, (2) review of the titles and abstracts to exclude clearly irrelevant articles, (3) in-depth full-text review of articles to determine inclusion, (4) extraction of relevant data from the included articles, and (5) synthesis of the extracted data and quality assessment.

### Database Search

The literature search for this study was conducted under another broad systematic review of predictors of different e-cigarette use patterns among young people, which were vaping initiation, escalation, continuation, prevalence, and vaping cessation.^21^ For this purpose, PubMed and PsycINFO databases were searched on June 19, 2022, using various combinations of subject headings, including Medical Subject Headings or MeSH terms and keywords such as “electronic cigarette,” “e-cigarette,” “vaping,” “vaping dependence,” “vaping initiation,” “vaping cessation,” “quit vaping,” “risk factors,” “predictors,” “correlates,” “association,” “adolescents,” “youth,” and “young adults” (see Supplementary Appendix 1). To capture machine learning-based studies predicting e-cigarette use patterns, we extended our search to the information technology database—IEEE Xplore on September 23, 2022. As machine learning is a novel statistical method used very recently in public health informatics,^22,23^ we included both conference papers and journal articles from the IEEE Xplore database to capture unpublished works. We also searched for gray literature in Google Scholar and the Ontario Tobacco Research Unit library databases in September 2022. All search results were further limited to English-language papers published within the year 2005 and 2022, as e-cigarettes first emerged in the European and American markets in 2006.^24^ For the customized Google Scholar search, it is recommended to go beyond the first 300 results to include gray literature in the review.^25^ Moreover, the literature search for this review was conducted as part of another broad systematic review, where we looked for articles on different e-cigarette use patterns. Hence, we screened the first 500 results through title and abstract screening. Finally, we updated all searches on July 15, 2023. The first 100 results of the most recently published articles from updated Google Scholar search were selected for title and abstract screening because we did not get any relevant article following this threshold. We also searched the reference lists of all included articles and subject matter experts (MC, PS, TJM, and DF) were consulted for potential papers. One reviewer (AK) conducted the database and gray literature search, and all citations were imported to the Covidence workflow platform, where duplicate papers were automatically removed.^26^

### Eligibility

In this review, we included studies that investigated associations between predictors and different vaping cessation-related behaviors such as intention to quit vaping, quit attempts, and vaping abstinence among young e-cigarette users. Hence, articles retrieved on other e-cigarette use patterns (vaping initiation, escalation, continuation, and vaping prevalence) under the broader systematic review were excluded.^21^ Although WHO has defined people between ages of 10 and 24 years as adolescents and young adults,^27^ several studies identified people up to age 35 years as young adults.^28–30^ Moreover, previous research has shown that quitting smoking by 34 years significantly lowers smoking mortality and morbidities.^31^ Hence, we decided to expand our target population to people between the ages of 10 and 35 years, so that the findings of this study could be applicable for a broader group of young population. We also included studies where the target population included people between ages of 10 and 35 years as subpopulation. The exclusion criteria were studies evaluating: (1) efficacy of cessation interventions; (2) predictors of smoking cessation or other tobacco products cessation among e-cigarette users; (3) predictors of cessation of cannabis vaping, dual use, or poly-substance use; (4) experimental studies, qualitative studies, literature reviews, commentaries, letters, protocols; and (5) animal studies. No studies were excluded based on sample size, follow-up duration, participant socioeconomic status, or country of origin.

### Study Selection

Two reviewers (AK and SS) independently screened each title and abstract followed by full texts of the remaining articles in accordance with the inclusion and exclusion criteria. Disagreements were resolved by discussions among the two reviewers (AK and SS) and final decision was taken often with the guidance from another reviewer (MC).

### Data Extraction

Two custom-made data extraction forms were developed, which included general characteristics of the included studies (author and year, country, sample source, study design, analytical sample size, participants’ age at baseline, participants’ vaping status at baseline, follow-up period and number of follow-ups after baseline, outcome, analytical method of prediction, and model adjustment) and identified predictors in the studies. By following the method used by Vallata et al. (2021)^32^ and Cengelli et al. (2012)^33^ studies, we extracted data on all predictors that were used in the analyses, their level of statistical significance (significant association, no association), and direction of the effect (positive, negative) as mentioned in any given study. We used *p* < .05 or in absence of *p* value, 95% confidence interval (CI) that did not include null value to measure statistical significance. In contrast to Vallata et al.,^32^ we have included all predictors in our review, even if the predictor was used in only one study. For some predictors, especially substance-use related predictors, we collapsed some categories and combined them. For example, “other tobacco products (OTPs) use” included tobacco use other than cigarettes and e-cigarettes including hookah, waterpipe, snus, smokeless tobacco, etc. While one reviewer (AK) independently extracted data from each article, another reviewer (SS) checked for accuracy of the extracted data. Any disagreements were resolved through discussions between reviewers.

### Data Synthesis

We categorized the outcomes in the studies of vaping cessation-related behaviors into three different categories: intention to quit vaping, quit attempts, or vaping abstinence. While intention to quit vaping was indicated by future intention to quit e-cigarette among users who were continuing vaping, quit attempts were measured among users who tried to quit previously. Vaping abstinence was defined by successfully quitting vaping for a defined time period such as 30 days, 6 months, or 12 months.

As in Vallata et al.,^32^ we analyzed the data for each individual predictor and categorized the predictors in descending order of frequency of reported statistical significance into four categories: (1) probable predictor (ie, if a predictor was found statistically significant by both studies when examined by only two studies or, in case of more than two studies, statistical significance was showed in at least 50% of the total studies); (2) possible predictor (ie, if found statistically significant by one study out of maximum two studies or, in case of more than two studies, the predictor was found statistically significant in at least 25%–49% of the total studies); (3) probably unrelated (ie, if no significant association were found when the predictor was tested by only two studies or, in case of more than two studies, found statistically significant in <25% of the total studies); and (4) insufficient evidence (ie, if the variable was examined by only one study and no significant association was detected). The final direction of effect (positive and negative) for each individual predictor was determined based on the direction of association observed in the majority of the studies. The predictors of positive direction were those which increased the probability of the outcomes, and the predictors of negative direction were those which lowered the probability of the outcomes. An additional category—“inconsistent direction” was added to the previous four categories to represent the predictors for which the number of articles reporting positive and negative associations were equal. We summarized these five groups of predictors for each of the three outcomes. Although our target population was between 10 and 35 years, there was considerable variability and overlapping between the target population of the included studies, with some using a very broad age range (eg, 15–36 years) for their target population. Hence, we made two to three categories of overlapping age groups and summarized the predictors of all three outcomes for individual age groups. Additionally, we also looked into whether the identified predictors differed between the cross-sectional and longitudinal studies. Data synthesis was conducted by one reviewer (AK) with guidance from other reviewers (MC, PS, and TJM).

### Quality Assessment

We used the widely accepted Quality in Prognosis Studies (QUIPs) tool,^34^ which is specifically designed for assessing bias of the predictive modeling studies. QUIPs^34^ includes six domains: study participation, study attrition, prognostic factor measurement, outcome measurement, study confounding, statistical analysis, and reporting. Each of these six domains consists of 3–7 items checklist and rating in each domain is provided as low, moderate, and high risk of bias. As domain two “study attrition” is based on adequate sample retention in the longitudinal studies, we rated this domain as “not applicable” for cross-sectional studies. Quality assessment was done independently by two reviewers (AK and SS) and any disagreements were resolved by discussions between the reviewers.

## Results

**Figure 1.**
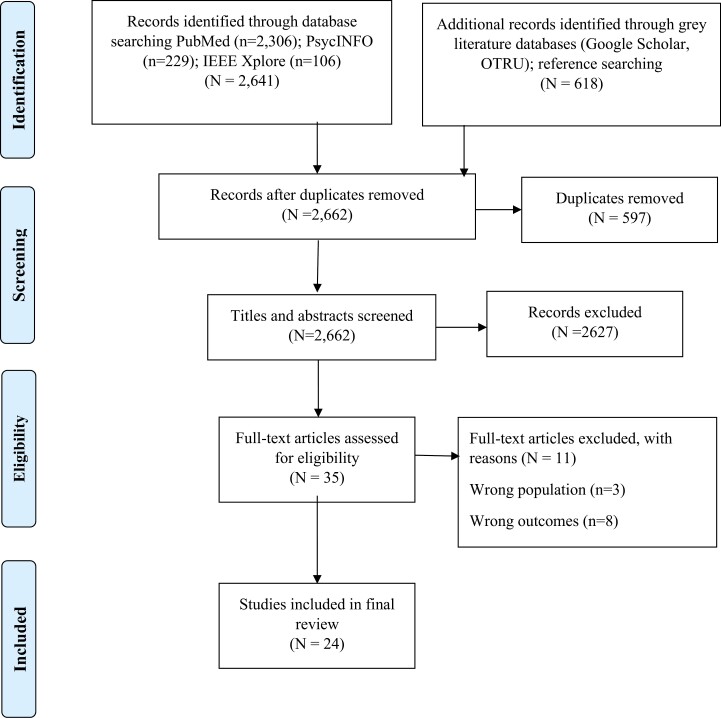
PRISMA flow diagram demonstrating selection of studies.

As the literature search was a part of the broad systematic review,^21^ we retrieved a total of 2641 articles from the academic databases and 618 articles from the gray literature search and reference searching of the included studies. Following the removal of 597 duplicates, we screened titles and abstracts of 2662 articles, of which 2627 articles were removed including studies on other e-cigarette use patterns (vaping initiation, escalation, continuation, and vaping prevalence) and 35 articles were selected for full-text screening. After removing 11 articles for not meeting the eligibility criteria for target population (*n* = 3) and outcomes (*n* = 8) (Figure 1), a total of 24 studies^35–58^ were included in the final review. The detailed selection process of the articles is presented in the PRISMA flow diagram (Figure 1).

Of the 24 studies,^35–58^ 70.8% (*n* = 17) were published within last 3 years, all of them were published within last 6 years. Most of the studies (87.5%, *n* = 21) were carried out in the United States, 54.2% (*n* = 13) had a sample size of 100–1000 and 41.7% (*n* = 10) had a sample size of 1001–5000 (Table 1). About 75% (*n* = 18) studies examined a population aged at least 18 years of age, and 54.2% (*n* = 13) studies examined adolescent populations aged below 18 years. One study^38^ examined the predictors of intention to quit vaping among the homeless population. The majority of the studies (70.8%, *n* = 17) were cross-sectional, while 29.2% (*n* = 7) were longitudinal studies. The longest duration a single study followed up the participants was 6 years. Logistic regression (75%, *n* = 18) was the most used method for prediction, and all studies adjusted their model for confounders.

**Table 1.** Summary Statistics of Included Studies (*N* = 24)

Characteristics	Number of papers (*n*)	Percentage (%)
Outcome		
Intention to quit	15	62.5
Quit attempts	11	45.8
Vaping abstinence	7	29.2
Years published		
2018–2020	7	29.2
2021–2023	17	70.8
Country		
The United States	21	87.5
Malaysia	1	4.2
Multicountry	2	8.3
Study design		
Longitudinal	7	29.2
Cross-sectional	17	70.8
Sample size		
100–1000	13	54.2
1001–5000	10	41.7
>5001	1	4.2
Sample age (years)		
<18	13	54.2
≥18	18	75
Analytical method for prediction		
Logistic regression	18	75
Linear regression	2	8.3
Hierarchical regression	3	12.5
Latent class analysis	2	8.3
Structural equation modeling	1	4.2
Adjusted for confounders		
Yes	24	100
No	0	0
No. of predictors investigated		
<10	13	54.2
10–20	10	41.7
>21	1	4.2

Out of 24 studies, 62.5% (*n* = 15) evaluated the predictors of intention to quit vaping, 45.8% (*n* = 11) investigated quit attempts, and 29.2% (*n* = 7) identified predictors of vaping abstinence. (Table 1). While five^40,44,50,52,54^ out of seven studies defined vaping abstinence by being abstinent for 30 days, one study measured 6-month vaping abstinence^56^ and another study measured 12-month vaping abstinence^57^ (Supplementary Appendix 2). Six out of seven longitudinal studies^40,50,52,54,56,57^ were on vaping abstinence, whereas the seventh study^42^ identified predictors of quit attempts.

### Quality Assessment

Of the 24 studies, all studies were rated to have low risk of bias in terms of prognostic factor measurement, and outcome measurement (see Supplementary Appendix 3). In general, studies were also found to have low risk of bias in the domain of study participation, study confounding, and statistical reporting and analysis. Only two studies^56,57^ on vaping abstinence had high risk of bias for study participation, and one study^43^ on quit attempts had moderate risk of bias for confounding. Two studies^52,53^ were rated to have high risk of bias for analysis and reporting, of which one identified predictors of intention to quit vaping and quit attempts,^53^ while another assessed predictors of vaping abstinence.^52^ Of the seven longitudinal studies, five had high risk of bias for study attrition,^42,50,52,56,57^ of which only one was on quit attempts,^42^ and the rest of the four studies were on vaping abstinence.^50,52,56,57^ Of the remaining two longitudinal studies, both assessing vaping abstinence, one had moderate risk of bias,^40^ and another had low risk of bias^54^ in the domain of study attrition (see Supplementary Appendix 3). None of the 24 studies had any association with tobacco or vaping industries.

### Predictors

Supplementary Appendix 4 presents the predictors identified by each individual study and Supplementary Appendix 5 demonstrates the process of determining the direction of association and category of the predictors for all three outcomes. We identified a total of 106 predictors, of which 84 were predictors of intention to quit vaping, 51 were predictors of quit attempts, and 47 were predictors of vaping abstinence. These predictors included a wide variety of factors including sociodemographics, e-cigarette use-related factors, dependence, psychosocial factors, peer, family, social factors, and policy-related factors. The total number of predictors examined by a single study ranged from 1 to 21, and the highest number of statistically significant predictors found by two studies^52,54^ was 10.

Of the 84 predictors of intention to quit vaping, 11 were “probable” predictors, 32 were “possible” predictors, 29 had “insufficient evidence,” 11 were “probably unrelated” and one had “inconsistent direction” (Table 2 and Supplementary Appendix 6). The “probable” predictors that increased the probability of intention to quit vaping were disposable e-cigarette device use, using e-cigarette because of peer or family vaping, high-harm perception of vaping, and exposure to anti-vaping contents. In contrast, higher frequency of vaping, current cigarette smoking, alcohol drinking, OTPs use, dual use, poly tobacco use, and having friends who vape were identified as “probable” predictors that decreased the probability of having intention to quit vaping.

**Table 2.** “Probable” and “Possible” Predictors of Intention to Quit Vaping, Quit Attempts and Vaping Abstinence

Direction of association	Intention to quit	Quit attempts	Vaping abstinence
“Probable” predictors			
Positive	Disposable EC device use, Reasons for EC use—peer/family vaping, High-harm perception of vaping, Exposure to anti-vaping contents.	Reasons for EC use—curiosity, High-harm perception of vaping, Current OTPs use.	High-harm perception of vaping.
Negative	Frequency of EC use, Current cigarette smoking, Current alcohol drinking, Current OTPs use, Current dual use, Current poly tobacco use, Peer vaping.	Race—White, Level of nicotine dependence, Frequency of EC use, Current dual use, Current poly tobacco use.	Older age, Sweet-flavored EC use, Level of nicotine dependence, Current OTPs use.
“Possible” predictors			
Positive	Sexual orientation—heterosexual, Higher education, COVID-19 outbreak, No anxiety, no fatigue, No neuroticism, Less cognitive control, Reasons for use—peer vaping, Unaware of flavor in EC, High nicotine concentration, Preparedness for quitting, Perceived benefits of cessation, Perceived COVID-19 threat of EC, Perceived COVID-19 related benefits of quitting, Parental monitoring and support, Increased age × homeless, Harm perception × homeless.	Sexual orientation—heterosexual, Migrant, High cravings, High nicotine concentration, EC device—unknown, Self-efficacy/confidence in quitting, Perceived COVID-19 threat of EC use, Perceived COVID-19 related benefits of quitting, Peer vaping.	Parental monitoring.
Negative	Gender identity—transgender or gender diverse, Married/living with partners, Reasons for use—discrete, Reasons for use—product features, Anxiety, Neuroticism, Fatigue, Current cannabis use, Seeing important people vaping, Social acceptability of vaping, EC accessibility, Health warnings in EC package, Withdrawal symptoms × homeless, Homeless × frequency of use, Reasons for use—socializing × homeless.	Duration of vaping (years), Reasons for use—discrete, Past quit attempts, Current cannabis use, Cravings × female sex.	Race—White, Parental education, Duration of vaping (years), Level of nicotine dependence, Frequency of vaping, Reasons for EC use—low cost, Cognitive enhancement from EC, EC device—open pods, Owning JUUL device, Flavored EC use, Menthol/mint flavored EC use, Nicotine vaping, nicotine content, Internalized problems, Current cigarette use, OTPs use Current alcohol use, Current cannabis use, Current illicit drug use, Family/household and peer vaping, Seeing important people vaping, Social media follower, EC price affordability.

COVID-19 = Coronavirus disease 2019; EC = e-cigarette; OTPs = other tobacco products.

Of the 51 predictors of quit attempts, eight were “probable” predictors, 14 were “possible” predictors, 15 had “insufficient evidence,” 10 were “probably unrelated” and four had “inconsistent direction” (Table 2 and Supplementary Appendix 6). The “probable” predictors that promote quit attempts were using e-cigarette because of curiosity, high harm perception of vaping, and current OTPs use, while being White, high level of nicotine dependence, high frequency of vaping, current dual use, and poly tobacco use lowered the probability of quit attempts.

Of the 47 predictors of vaping abstinence, five were “probable” predictors, 25 were “possible” predictors, 13 had “insufficient evidence,” three were “probably unrelated,” and one had “inconsistent direction” (Table 2 and Supplementary Appendix 6). The “probable” predictor that facilitated vaping abstinence was high harm perception of vaping, while older age, high level of nicotine dependence, sweet-flavored e-cigarette use, and current OTPs use acted as barriers of vaping abstinence.

### Predictors by Different Age Groups and Study Designs

Supplementary Appendix 7 showed the distribution of the predictors for all three outcomes by different age groups. Studies examining intention to quit vaping were categorized into three overlapping age groups of ~10–19 years (*n* = 6, 40%), ~13–25 years (*n* = 7, 46.7%), and ~15–35 years (*n* = 2, 13.3%). High harm perception of vaping was detected as a “probable” predictor that increased the probability of intention to quit vaping for both ~10–19 years and ~13–25 years age groups, whereas increased frequency of vaping was found as the only “probable” predictor among ~15–35 years age group that lowered this probability. Additionally, being Hispanic was found to increase the intention to quit vaping among ~13–25 years age people.

Studies on quit attempts were categorized into three overlapping age groups of ~10–19 years (*n* = 7, 63.6%), ~18–25 years (*n* = 2, 18.2%), and ~15–35 years (*n* = 2, 18.2%). No “probable” predictors were detected for ~18–25 years age group, while being White, and increased frequency of vaping were detected as “probable” barriers of quit attempts among ~15–35 years age group. Predictors identified for ~10–19 years age group were almost similar to the overall “probable” predictors of quit attempts (Table 2).

Studies on vaping abstinence were divided into two overlapping age groups of ~10–19 years (*n* = 4, 57.1%) and ~18–29 years (*n* = 3, 42.9%). While older age was identified as the only “probable” barrier of abstinence for ~10–19 years age group, those for ~18–29 years age group were higher level of nicotine dependence and sweet-flavored e-cigarette use (Supplementary Appendix 7).

Supplementary Appendix 8 presents the comparison of predictors between cross-sectional and longitudinal studies for all three outcomes. While the majority of the studies on intention to quit (*n* = 14, 93.3%) and quit attempts (*n* = 10, 91%) were cross-sectional studies, most of the studies on vaping abstinence (*n* = 6, 85.7%) were longitudinal studies (Supplementary Appendix 2). Hence, the predictors of intention to quit and quit attempts detected by cross-sectional studies were disproportionately higher compared to those detected by longitudinal studies. On the contrary, the predictors of vaping abstinence detected by longitudinal studies were substantially higher than those detected by cross-sectional studies. Moreover, no “probable” predictors were detected by the longitudinal studies on intention to quit and quit attempts and by cross-sectional studies examining vaping abstinence (Supplementary Appendix 8).

## Discussion

In this review, we identified a wide variety of factors that predict three types of vaping cessation related behaviors among young people and grouped them into “probable,” “possible,” “insufficient evidence,” “probably unrelated,” and “inconsistent direction” categories. Most of the studies examined predictors of intention to quit vaping and quit attempts, only one-third of the studies focused on vaping abstinence or quit success. Across the three outcomes, the identified “probable” predictors encompassed demographics, reasons for using e-cigarette, e-cigarette use pattern-related factors, harm perception of vaping, level of nicotine dependence, peer/family-related factors, OTPs and other substance use-related factors, and exposure to anti-vaping contents (Table 2 and Supplementary Appendix 6). While the “probable” predictors are important to identify the high-risk population who need targeted intervention for increasing their likelihood of having intention to quit vaping, and quit attempts as well as being abstinent, the “possible,” “insufficient evidence,” and “inconsistent direction” predictors need further testing by future research.

While comparing the “probable” predictors between different vaping cessation related behaviors, we found that having high harm perception of vaping increased the probability of intention to quit vaping, quit attempts, and ultimately being successfully abstinent. This finding matched with previous research findings on smoking cessation.^59,60^ However, previous research showed that e-cigarette users were twice as likely to perceive an e-cigarette as safer and less harmful than tobacco cigarettes compared to nonusers among young people.^61^ A principal reason behind low-risk perception of e-cigarettes among youth is related to a misperception about the ingredients of e-liquids and uncertainty regarding the long-term side effects (ie, cancer) of vaping.^62^ Hence, in addition to conducting future longitudinal research on the long-term side-effects of using e-cigarettes, new programs to raise awareness regarding the potential health risks of nicotine vaping should be undertaken at the school and community level (ie, the “Real Cost” youth e-cigarette prevention campaign^63^).

Another “probable” predictor, current OTPs use, was found to decrease the probabilities of intention to quit vaping and being abstinent but increase the probability of making a quit attempt. Similar findings were found in smoking cessation research,^64^ where it is suggested that OTPs are attractive to smokers to make a quit attempt, but not always associated with successful quitting. We also found that current dual use of cigarettes and e-cigarettes and poly tobacco use lower the probabilities of intention to quit vaping and quit attempts (Table 2). Previous research suggests that dual users are less inclined to quit e-cigarette because of lower harm perception of vaping.^65^ However, both current cigarette smoking and current OTPs use were “possible” negative predictors of vaping abstinence indicating that dual users and poly tobacco users had lower success rate in quitting (Table 2), which is supported by previous research.^65^ Therefore, further investigations focusing on quitting smoking and OTPs before attempting to quit vaping among these populations should be explored in future. Among demographic factors, being White came out as a barrier of quit attempt and a “possible” negative predictor of being abstinent. These findings contradict our knowledge from smoking cessation^66,67^ and the reason behind this is not clear. However, similar to smoking cessation research, we found that younger people might have higher likelihood of successful quitting than older individuals.^68^

Consistent with previous evidence on smoking cessation,^69,70^ we found that level of nicotine dependence negatively impacted making quit attempts and vaping abstinence. Similarly, frequency of e-cigarette uses also decreased probabilities of intention to quit smoking and quit attempts (Table 2). These findings can guide vaping cessation approaches like tapering use of e-cigarettes or “cold turkey” methods and might increase the probability of successful quitting. Evidence in support of using nicotine replacement therapy (NRT) and non-NRT are not well established yet.^71^ So, in addition to evaluating the effectiveness of pharmacotherapy for vaping cessation by future research, cessation programs should explore other behavioral interventions. Among other “probable” predictors, disposable e-cigarette device use increased the likelihood of intention to quit vaping, but using a sweet flavor in e-cigarettes lowered the likelihood of being abstinent (Table 2). These findings have implications for public health policy. Health Canada recently proposed a ban of flavors other than tobacco and mint/menthol in vaping products.^72^ Our findings suggest that this regulation should be effective in lowering the prevalence of vaping among young people. Additionally, we found that those who were using e-cigarettes because of peer or family vaping or out of curiosity had higher probabilities of having intention to quit vaping and making quit attempts. But e-cigarette use among friends was found to lower the likelihood of having intention to quit. This finding match with our knowledge from predictors of smoking cessation, where smokers with higher number of smoking friends were less likely to intend to quit.^73^ However, none of these factors promote vaping abstinence in our review. Overall, this indicates that for successful quitting, factors other than reasons for e-cigarette use, such as harm perception of vaping, level of vaping dependence, and OTPs use may have more significance.

Our age-based analysis of the predictors in Supplementary Appendix 7 clearly showed that studies varied widely in their definition of young people and investigated predictors. Because of these wide variations and the low number of studies found for certain age groups, these findings did not reveal any useful information other than the fact that high harm perception of vaping mainly increased the probabilities of intention to quit vaping and quit attempts among younger e-cigarette users (~10–19 years), while dual use and poly tobacco use diminished such probabilities. In this review, we also found that predictive modeling studies in e-cigarette research are still new and out of the 24 included studies, only seven studies were conducted with longitudinal study design. As exposures precede outcomes in longitudinal studies, these studies were more likely to suggest cause-and-effect relationships than cross-sectional studies.^74^ Moreover, tracking natural history of vaping cessation behaviors, particularly investigating number of quit attempts before successful abstinence requires longitudinal research. While the predictors of vaping abstinence were mostly detected by the longitudinal studies (*n* = 6), those of intention to quit and quit attempts were detected by mainly cross-sectional studies (Supplementary Appendix 8). Hence, considering the low number of studies, more prospective longitudinal research should be conducted to further identify and explore all three outcomes. Most of the included studies were rated as having low risk of bias in almost all domains. However, the majority of the longitudinal studies had high risk of bias in the study attrition domain. Hence, it is important that future researchers should be open and follow the guidelines regarding reporting data of the participants who were lost to follow-up. Almost 90% of the studies included in this review were conducted in the U.S. population. This is important to consider because sociocultural environments as well as regulatory policies regarding e-cigarette use among young population differ between different jurisdictions.^75^ Therefore, more research should be conducted in other non-U.S. jurisdictions to understand how different policies affect vaping cessation behaviors.

### Strengths and Limitations

We conducted a comprehensive review summarizing predictors of three different vaping cessation behaviors among young people. Although there are some similarities with the systematic review of Amin et al. (2023)^18^ our review has provided some distinguishable findings. While Amin et al.^18^ did not include any cross-sectional studies in their review, we demonstrated that intention to quit vaping and quit attempts were mainly investigated by cross-sectional studies. Moreover, among the longitudinal studies included in our review, only one overlapped with their review.^50^ Additionally, all longitudinal studies included in the review of Amin et al.^18^ had a target population of ≥18 years, whereas we have included studies that focused on adolescent population of ~10–19 years of age. Hence, readers should keep these differences in mind while comparing our findings with their review. Nevertheless, our findings should be considered with a few limitations. First, we did not conduct any meta-analysis of our data. Rather than focusing on association with a single predictor, our main objective was to summarize the predictors including significant ones, nonsignificant ones, and those that need further research. Second, we defined any specific time period of abstaining from using e-cigarette as vaping abstinence. However, previous research revealed that the predictors of short-term smoking abstinence (ie, 30 days) differed from those of long-term (ie, 12 months) smoking abstinence (eg, age, heaviness of smoking, and level of dependence).^76,77^ Only two studies^56,57^ in our review met the criteria of long-term abstinence (6–12 months),^77,78^ and identified flavors of e-cigarette and current cigarette smoking as negative “possible” predictors (Table 2 and Supplementary Appendix 4). Therefore, rather than reporting them separately, we presented the predictors of both short-term and long-term abstinence under one outcome, although future research should separate these two outcomes and examine the similarities and differences between their predictors. Third, our findings are not generalizable for all types of young population. For example, we did not find any study investigating predictors of vaping cessation-related behaviors among pregnant females. The predictors influencing young pregnant populations’ behavior might be different from general population, hence should be emphasized while doing sample selection. Fourth, we acknowledge that we considered a wide age group (10–35 years) as the target population. Even after dividing this population into overlapping age groups, some of these age groups were very broad (eg, 15–35 years). One of the reasons behind it was including studies that used a broad age range for their target population.^38,45,46,54^ Hence, future studies should look for predictors of vaping cessation-related behaviors among adolescents (10–19 years) and young adults (20–35 years) separately. Fifth, we excluded experimental studies from this review, so we cannot comment on the effectiveness of vaping cessation interventions. As we targeted to identify the individual, social, and environmental predictors influencing vaping cessation which are difficult to measure in a controlled environment,^79^ we limited our review to nonexperimental studies. Sixth, we excluded non-English literatures, which might lead to missing studies published in other languages. Conducting another review looking for studies published in non-English language might complement our findings.

## Conclusions

We found several individual and environmental factors that most “probably” were associated with intention to quit vaping, quit attempts, and vaping abstinence, which have both public health and policy implications. Our findings may be used to plan targeted vaping intervention programs for high-risk populations, investigate causal associations between these “probable” predictors and outcomes, and reexamine potential associations with the predictors that need further testing. Improving public awareness of the harm associated with vaping and promoting cessation of cigarettes and other tobacco products among concurrent users are crucial steps to increase intention to quit and encourage quit attempts among the younger population. Finally, predictive modeling studies investigating vaping cessation among young people are still new. While there is need to conduct more prospective longitudinal research on all three outcomes, exploring predictors of both short-term and long-term vaping abstinence demands further attention to develop more targeted public health interventions. Additionally, researchers should consider conducting research in different jurisdictions and population groups, which includes investigating adolescents and young adults separately to better understand the factors that influence young people’s vaping cessation behaviors.

## Supplementary Material

Supplementary material is available at *Nicotine and Tobacco Research* online.

ntae181_suppl_Supplementary_Materials

## Data Availability

Data are available in the body of the manuscript, Supplementary Materials, and in original cited works.
