# Supplementary material for: A Systematic Review of Predictors of Vaping Cessation Among Young People
Source: Nicotine Tob Res. 2024 Jul 20;27(2):169–78. doi: 10.1093/ntr/ntae181 (PMC11750738; doi:10.1093/ntr/ntae181)
Supplement: ntae181_suppl_Supplementary_Materials [file ntae181_suppl_supplementary_materials.docx]

**Supplementary Materials**

**A systematic review of predictors of vaping cessation among young people**

*Appendix 1. Database search strategies*

*Appendix 2. General characteristics of all included articles (N=24).*

*Appendix 3. QUIPS Risk of bias assessment of the included studies (N=24).*

*Appendix 4. Predictors identified from all included studies (N=24).*

*Appendix 5. Directions of the associations of predictors of intention to quit, quit attempts, and vaping abstinence.*

*Appendix 6. ‘Insufficient evidence’, ‘probably unrelated’ and ‘inconsistent direction’ predictors of intention to quit, quit attempts and vaping cessation.*

*Appendix 7. Summary of predictors of intention to quit vaping, quit attempts, and vaping abstinence by different age groups.*

*Appendix 8. Distribution of predictors by cross-sectional and longitudinal studies.*

***Appendix 1. Database search strategies***

**PubMed Search strategy**

Date: June 19, 2022

| Searches | Results |
| --- | --- |
| 1. "vaped"[All Fields] OR "vaping"[MeSH Terms] OR "vaping"[All Fields] OR "vapes"[All Fields] OR "e cigarette*"[All Fields] OR "electronic cigarette*"[All Fields] OR "electronic nicotine delivery system"[All Fields] OR "vaping dependen*"[All Fields] OR "vaping initiation"[All Fields] OR "vaping cessation"[All Fields] OR "quit vaping"[All Fields] | 9,513 |
| 2. "risk factors"[All Fields] OR "predictor*"[All Fields] OR "correlate*"[All Fields] OR "associat*"[All Fields] | [6,955,](https://pubmed.ncbi.nlm.nih.gov/?term=%22risk+factors%22+OR+predictor%2A+OR+correlate%2A+OR+associat%2A&sort=relevance)862 |
| 3. "adolescent"[MeSH Terms] OR "adolescent"[All Fields] OR "adolescence"[All Fields] OR "adolescences"[All Fields] OR "adolescents"[All Fields] | 2,294,588 |
| 4. "young adult"[MeSH Terms] OR "youth"[All Fields] OR "young adult"[All Fields] | [1,109,15](https://pubmed.ncbi.nlm.nih.gov/?term=%22young+adult%22%5BMeSH+Terms%5D+OR+%22youth%22%5BAll+Fields%5D+OR+%22young+adult%22%5BAll+Fields%5D&sort=)2 |
| 5. ("adolescent"[MeSH Terms] OR "adolescent"[All Fields] OR "adolescence"[All Fields] OR "adolescences"[All Fields] OR "adolescents"[All Fields]) OR ("young adult"[MeSH Terms] OR "youth"[All Fields] OR "young adult"[All Fields]) | 2,798,250 |
| 6. ("vaped"[All Fields] OR "vaping"[MeSH Terms] OR "vaping"[All Fields] OR "vapes"[All Fields] OR "e cigarette*"[All Fields] OR "electronic cigarette*"[All Fields] OR "electronic nicotine delivery system"[All Fields] OR "vaping dependen*"[All Fields] OR "vaping initiation"[All Fields] OR "vaping cessation"[All Fields] OR "quit vaping"[All Fields]) AND ("risk factors"[All Fields] OR "predictor*"[All Fields] OR "correlate*"[All Fields] OR "associat*"[All Fields]) AND (("adolescent"[MeSH Terms] OR "adolescent"[All Fields] OR "adolescence"[All Fields] OR "adolescences"[All Fields] OR "adolescents"[All Fields]) OR ("young adult"[MeSH Terms] OR "youth"[All Fields] OR "young adult"[All Fields])) | 2,272 |
| 7. Search: #6 Filters: English, Humans, from 2005/1/1 - 2022/6/19 | 1,950 |

Updated search on July 15, 2023: additional results (n=356)

**Search strategy of PsycINFO**

Date: June 19, 2022

| **Searches** | **Results** |
| --- | --- |
| 1. exp Electronic Cigarettes/ | 2,246 |
| 2. ((vaping or e-cigarette*) adj2 (dependen* or initiation or susceptibility or cessation or quit)).tw. | 313 |
| 3. ((vaping or e-cigarette*) adj2 (dependen* or initiation or susceptibility or cessation or quit)).ti. | 63 |
| 4. exp risk factors/ | 92,275 |
| 5. exp prediction/ | 29,904 |
| 6. ((risk* or predict* or correlate* or associat*) adj2 factor*).tw. | 171,459 |
| 7. ((risk* or predict* or correlate* or associat*) adj2 factor*).ti. | 27,382 |
| 8. exp adolescent behavior/ | 3,378 |
| 9. exp emerging adulthood/ | 5,330 |
| 10. (youth* or adolescen* or (young adj2 adult*)).tw. | 390,154 |
| 11. (youth* or adolescen* or (young adj2 adult*)).ti. | 199,780 |
| 12. 1 or 2 or 3 | 2,302 |
| 13. 4 or 5 or 6 or 7 | 222,012 |
| 14. 8 or 9 or 10 or 11 | 392,035 |
| 15. 12 and 13 and 14 | 207 |
| 16. limit 14 to (human and english language and yr="2005 -Current") | 191 |

Updated search on July 15, 2023: additional results (n=38)

**IEEE Xplore search**

Date: September 23, 2022

Search terms: ("Full Text & Metadata":vaping OR "Full Text & Metadata":e-cigarette OR "Full Text & Metadata":electronic cigarette) AND ("Full Text & Metadata":predict OR "Full Text & Metadata":risk factor OR "Full Text & Metadata":associated) AND ("Full Text & Metadata":Adolescent OR "Full Text & Metadata":youth OR "Full Text & Metadata":young adult)

Filters applied: Conferences, Journals, 2005-2022

Results: 79

Updated search on July 15, 2023: additional results (n=27)

**Google Scholar search**

Date: September 23, 2022

Search terms: (e-cigarette OR vaping) ('risk factor' OR predictor OR associated) (adolescent OR youth OR young adult)

Filters applied: 2005-2022

Results: 17,100 (selected first 500 to screen)

Updated search on July 15, 2023: additional results (n=9160) (selected first 100 to screen)

**Ontario Tobacco Research Unit (OTRU) library search**

Date: September 23, 2022

Search terms: (e-cigarette OR vaping) ('risk factor' OR predictor OR associated) (adolescent OR youth OR young adult)

Filters applied: 2005-2022

Results: 16

Updated search on July 15, 2023: additional results (n=0)

***Appendix 2. General characteristics of all included articles (N=24).***

| **Author and year** | **Country** | **Sample source** | **Study design** | **Analytical sample size; participant age at baseline** | **Participants’ vaping status at baseline** | **Follow-up period; number of follow-ups after baseline** | **Outcome(s)** | **Analytical Method of prediction** | **Adjustment** |
| --- | --- | --- | --- | --- | --- | --- | --- | --- | --- |
| Alalwan et al., 2022^50^ | The US | University-based | Longitudinal | 225; ~18-19 years | Past 30-day JUUL users | 2 years; 2 | Self-reported 30-day vaping abstinence; intention to quit | Logistic regression | Yes |
| Romm et al., 2022^42^ | The US | PATH (Population-based) | Longitudinal | 256; 12-16 years | Mixed | 1 year; 1 | Quit attempts | Latent class analysis followed by Latent transition analysis | Yes |
| Ahuja et al., 2022^39^ | The US | PATH (Population-based) | Cross-sectional | 349; 12-17 years. | Past 30-day exclusive e-cigarette users | NA | Intention to quit | Logistic regression | Yes |
| Ahuja et al., 2022 (a)^40^ | The US | PATH (Population-based) | Longitudinal | 243; 12-17 years | Past 30-day exclusive e-cigarette users | 1 year; 1 | Self-reported 30-day vaping abstinence | Logistic regression; SEM | Yes |
| Berg et al., 2021^46^ | The US | VAPES  (Population-based) | Cross-sectional | 1133; 18-34 years | Past 30-day e-cigarette users | NA | Intention to quit; past-year quit attempts | Logistic regression | Yes |
| Jun et al., 2022^41^ | The US | Population-based | Cross-sectional | 512;18-25 years | Past 30-day e-cigarette users | NA | Intention to quit; quit attempts | Hierarchical regression analysis | Yes |
| Saminathan et al., 2019^44^ | Malaysia | School-based | Cross-sectional | 1,285; 10-19 years | Ever users | NA | Self-reported 30-day vaping abstinence | Logistic regression | Yes |
| Dai, 2021^58^ | The US | NYTS (School-based) | Cross-sectional | 1,660; 11-18 years | Past 30-day users | NA | Intention to quit; past-year quit attempts; frequency of quit attempts | Logistic regression; linear regression | Yes |
| Chesaniuk et al., 2019^43^ | The US | MTurk survey (population-based) | Cross-sectional | 304; 18-24 years | Regular users | NA | Quit attempts | Logistic regression | Yes |
| Jankowski et al., 2019^48^ | Belarus, Lithuania, Poland, Russia, Slovakia | School-based | Cross-sectional | 2,061; mean age 20.4±2.2 | Mixed | NA | Intention to quit; quit attempts | Logistic regression | Yes |
| Kreslake et al., 2022^36^ | The US | Population-based | Cross-sectional | 1,007; 15-24 years | Past 30-day users | NA | Intention to quit | Logistic regression | Yes |
| Manning et al., 2021^35^ | The US | School-based | Cross-sectional | 685; 18-24 years | Past 30-day users | NA | Intention to quit | Hierarchical regression analysis | Yes |
| Cuccia et al., 2021^45^ | The US | TLC (Population-based) | Cross-sectional | 1,158; 15-36 years | Past 30-day users | NA | Intention to quit; past-year quit attempts | Logistic regression | Yes |
| Tucker et al., 2020^38^ | The US | Homeless locality based | Cross-sectional | 113; 13-25 years | Past 30-day users | NA | Intention to quit | Linear regression | Yes |
| Wackowski et al., 2019^37^ | The US, Canada, The UK | ITC (Population-based) | Cross-sectional | 12,064; 16-19 years | Mixed | NA | Intention to quit | Logistic regression | Yes |
| Zhang et al., 2020^49^ | The US | NYTS (School-based) | Cross-sectional | 1,769; 11-18 years | Past 30-day users | NA | Intention to quit; past year quit attempts | Logistic regression | Yes |
| Pulvers et al., 2021^47^ | The US | School-based | Cross-sectional | 133; mean age 20.1 ± 1.9 years | Past 30-day JUUL users | NA | Quit attempts | Logistic regression | Yes |
|  | The US | TATAMS (school-based) | Cross-sectional | 131; mean age ~12-17 years | Past 30-day users | N/A | Intention to quit; past-year quit attempts | Logistic regression | Yes |
| Steeger et al., 2022^57^ | The US | PATH (Population-based) | Longitudinal | 623; 18-24 years | Past 30-day users | 2 years; 1 | Self-reported 12-month vaping abstinence | Logistic regression | Yes |
| Yang et al., 2022^56^ | The US | H&H  (School-based) | Longitudinal | 1,977; 14-15 years | Mixed | 2 years; 4 | Self-reported 6- month vaping abstinence | Logistic regression | Yes |
| Sharma et al., 2022^55^ | The US | Population-based | Cross-sectional | 619; 18-24 years | Mixed | N/A | Intention to quit | Hierarchical regression | Yes |
| Pokhrel et al., 2023^54^ | The US | School-based | Longitudinal | 435; 18-29 years | Past 30-day users | 1 year; 1 | Self-reported 30 days vaping abstinence | Logistic regression | Yes |
| Lindpere et al., 2023^53^ | The US | NYTS (School-based) | Cross-sectional | 1,769; 11-18 years | Past 30-day users | N/A | Intention to quit; past year quit attempts | Logistic regression | Yes |
| Huang et al., 2023^52^ | The US | PATH (Population-based) | Longitudinal | 1,636; 12-17 years | Mixed | 6 years; 4 | Self-reported 30-day vaping abstinence | Latent class analysis | Yes |

Abbreviation: EC, e-cigarette; NA, not applicable; H&H, the Happiness and Health study; PATH, The Population Assessment of Tobacco and Health Study; TLC, The Truth Longitudinal Cohort; VAPES, The Vape shop Advertising, Place characteristics, Effects Surveillance study; NYTS, National Youth Tobacco Survey, ITC, International Tobacco Control Policy Evaluation Project.

***Appendix 2. QUIPS Risk of bias assessment of the included studies (N=24).***

| **Author and year** | **Study participation** | **Study attrition** | **Prognostic Factor Measurement** | **Outcome measurement** | **Study Confounding** | **Statistical Analysis and Reporting** |
| --- | --- | --- | --- | --- | --- | --- |
| Alalwan et al., 2022^50^ | Low | High | Low | Low | Low | Low |
| Romm et al., 2022^42^ | Low | High | Low | Low | Low | Low |
| Ahuja et al., 2022^39^ | Low | N/A | Low | Low | Low | Low |
| Ahuja et al., 2022 (a)^40^ | Low | Moderate | Low | Low | Low | Low |
| Berg et al., 2021^46^ | Low | N/A | Low | Low | Low | Low |
| Jun et al., 2022^41^ | Low | N/A | Low | Low | Low | Low |
| Saminathan et al., 2019^44^ | Low | N/A | Low | Low | Low | Low |
| Dai, 2021^58^ | Low | N/A | Low | Low | Low | Low |
| Chesaniuk et al., 2019^43^ | Low | N/A | Low | Low | Moderate | Low |
| Jankowski et al., 2019^48^ | Low | N/A | Low | Low | Low | Low |
| Kreslake et al., 2022^36^ | Low | N/A | Low | Low | Low | Low |
| Manning et al., 2021^35^ | Low | N/A | Low | Low | Low | Low |
| Cuccia et al., 2021^45^ | Low | N/A | Low | Low | Low | Low |
| Tucker et al., 2020^38^ | Low | N/A | Low | Low | Low | Low |
| Wackowski et al., 2019^37^ | Low | N/A | Low | Low | Low | Low |
| Zhang et al., 2020^49^ | Low | N/A | Low | Low | Low | Low |
| Pulvers et al., 2021^47^ | Low | N/A | Low | Low | Low | Low |
| Case et al., 2018^51^ | Low | N/A | Low | Low | Low | Low |
| Steeger et al., 2022^57^ | High | High | Low | Low | Low | Low |
| Yang et al., 2022^56^ | High | High | Low | Low | Low | Low |
| Sharma et al., 2022^55^ | Low | N/A | Low | Low | Low | Low |
| Pokhrel et al., 2023^54^ | Low | Low | Low | Low | Low | Low |
| Lindpere et al., 2023^53^ | Low | N/A | Low | Low | Low | High |
| Huang et al., 2023^52^ | Low | High | Low | Low | Low | High |

Abbreviation: N/A, not applicable; QUIPS, Quality in Prognosis Studies tool.

***Appendix 3. Predictors identified from all included studies (N=24).***

| **Author and year** | **Predictors** |
| --- | --- |
| Alalwan et al., 2022^50^ | Vaping abstinence: freshmen > juniors- younger age (P), Cigarillo users (N), Non-heavy users-nicotine dependence (P), Owning a JUUL device (N), Higher WSWS (Wisconsin Smoking Withdrawal Scale) score (N).  Quit intention: freshmen > juniors- younger age (P), baseline cigarillo users- OTPs users (N) |
| Romm et al., 2022^42^ | Black (P), Hispanic (P), (age, sex, parental education) NS |
| Ahuja et al., 2022^39^ | Parental support/talked about not using EC (P), Believing EC is harmful (P), Parents upset on finding out about EC use -parental monitoring (P), Peer vaping (N), Seeing important people vape (N), Important peoples' view on EC- social acceptability(N ), EC accessibility (N), Exposure to health warnings in EC package (P), Exposure to anti-tobacco campaign (IC), (Sex, race, ethnicity, nicotine dependence, nicotine awareness, Perceived addictiveness of EC, EC marketing exposure, getting EC coupons/exemplars/free samples) NS |
| Ahuja et al., 2022 (a)^40^ | Younger age group (P),  Location,  OTPs use (N),  Harm perception of EC-high (P),  Family vaping (N),  Seeing important people vaping (N),  (Sex, race, ethnicity, social acceptability of vaping, EC marketing exposure, getting EC coupons/exemplars/free samples, Exposure to health warnings in EC package, Exposure to anti-tobacco campaign) NS |
| Berg et al., 2021^46^ | Intention to quit:  Heterosexual (P),  Black (P),  Married/living with partner (N),  Frequency of use (N),  High nicotine content (P),  Using open system vaping device (N),  Cannabis use (N),  (age, sex, ethnicity, OTPs use) NS  Quit attempts:  Heterosexual (P),  Black (P),  Hispanic (P),  Frequency of use (N),  High nicotine content (P),  Using open system vaping device (N),  OTPs use (P)  Cannabis use (N),  (age, sex, Married/living with partner) NS. |
| Jun et al., 2022^41^ | Intention to quit:  Harm perception (P),  Perceived benefit of cessation (P),  Self-efficacy to quit (N),  Smoking (N),  OTPs use (IC),  Perceived COVID-19 threat of EC use (P),  Perceived COVID-19 benefit of quitting (P),  (Perceived barrier to cessation, EC dependence) NS  Quit attempts:  Harm perception (N),  EC dependence (N),  Self-efficacy to quit (P),  OTPs use (P),  Perceived COVID-19 threat of EC use (P),  Perceived COVID-19 benefit of quitting (P),  (Perceived benefit of cessation, perceived barrier to cessation, smoking) NS |
| Saminathan et al., 2019^44^ | Age (13-19 years> less than 13 years) (P),  Non-nicotine vaping (P),  Affordability (N),  (Sex, urbanicity, age of initiation, smoking, harm perception, Liking flavour of EC, Exposure to anti-vaping campaigns) NS. |
| Dai, 2021^58^ | Intention to quit:  Male sex (P),  EC device-modifiable system use (N),  Dual users or poly tobacco users (N),  Harm perception (P),  Reasons for EC use- peer vaping (P),  Reasons for EC use- discrete (N),  Cognitive impairment (P),  Exposure to anti-vaping campaigns (P),  (Age/school grade, race/ethnicity, sexual orientation, Frequency of EC use, vaping duration, Flavoured EC use, nicotine dependence, Exposure to EC marketing, reasons for EC use- quit smoking or OTPs or out of curiosity, household tobacco use, migrants) NS  Past-year quit attempts:  Migrants (P),  EC device type-unknown (P),  Dual users or poly tobacco users (N),  Harm perception (P),  Reasons for EC use- curiosity (P),  Reasons for EC use- discrete (N),  (Age/school grade, sex, race/ethnicity, sexual orientation, Frequency of EC use, vaping duration, Flavoured EC use, nicotine dependence, Exposure to EC marketing, reasons for EC use- peer vaping or quit smoking or OTPs, household tobacco use, cognitive impairment, exposure to anti-vaping campaigns) NS  Frequency of quit attempts:  Male sex (P),  Vaping duration >1 year (N),  EC device type-refillable (N),  Nicotine dependence (N),  (Others) NS |
| Chesaniuk et al., 2019^43^ | Race-White (N),  Tolerance, loss of control- Dependence (P),  Craving (P),  Carving x female sex (N),  (Sex, Frequency of use, smoking, Flavour, Affective enhancement, affective enhancement x sex, cognitive enhancement, dependence x sex) NS. |
| Jankowski et al., 2019^48^ | Intention to quit:  Increased age (P),  (Sex, breathing problem, medical vs non-medical studies) NS  Quit attempts:  (Age, sex, breathing problem, medical vs non-medical studies) NS |
| Kreslake et al., 2022^36^ | Harm perception (P),  Non-Hispanic Black (P)-> Non Hispanic White (N),  COVID-19 outbreak (P),  (Age, sex, income) NS |
| Manning et al., 2021^35^ | Smoking (N),  Alcohol use (N),  Race-White (N),  Neuroticism (N),  Anxiety sensitivity (N),  Fatigue (N),  (age, cannabis use, education, anxiety sensitivity x fatigue) NS |
| Cuccia et al., 2021^45^ | Intention to quit:  Frequency of use (N),  Harm perception (P),  Dependence (P),  (Age, sex, race, ethnicity, income, smoking, peer vaping) NS  Quit attempts:  Male sex (P),  Black (P)-> White (N),  Non-Hispanic (P),  Frequency of use (N),  Harm perception (P),  Dependence (P),  Peer vaping (P),  (Age, income, smoking) NS |
| Tucker et al., 2020^38^ | Homeless x Older age (P),  Homeless x Withdrawal symptoms (N),  Homeless x frequency of use (N),  Homeless x harm perception (P),  Homeless x reasons for use-socializing (N)  Homeless x (Sex, race, ethnicity, sexual orientation, sleeping outside, stress, seeing others use, Reasons for use- discrete or quitting smoking or flavour or nicotine content or good psychoactive effect) NS |
| Wackowski et al., 2019^37^ | Exposure to anti-vaping campaign (P),  (Exposure to pro-vaping content or mixed information content or unknown, age, sex, race, ethnicity, location, past 30-day e-cigarette use, exposure to EC marketing) NS |
| Zhang et al., 2020^49^ | Intention to quit:  Male sex (P),  Perceived flavour use in e-cigarette- unknown (P),  Dual use or Polytobacco use (N),  Harm perception (P),  (Age, race, ethnicity, dependence, household tobacco use, exposure to EC marketing) NS  Quit attempts:  Black (P),  Non-Hispanic (P),  Hispanic (P),  Dependence (P),  Dual use or polytobacco use (N),  Harm perception (P),  (Age, sex, household tobacco use, exposure to EC marketing, Perceived flavour use in e-cigarette) NS |
| Pulvers et al., 2021^47^ | Male sex (N),  OTPs use- hookah, shisha and waterpipe use (N),  Dependence (N),  Past quit attempts (N),  (Age, sexual orientation, race, ethnicity, income, smoking, non-disposable ENDS use) NS |
| Case et al., 2018^51^ | Intention to quit:  Dual users (N),  Nicotine dependence (N),  (Age, sex, race/ethnicity) NS.  Quit attempts:  Dual users (N),  Nicotine dependence (N),  Male sex (N),  (Age, race/ethnicity) NS. |
| Steeger et al., 2022^57^ | Any type of flavoured EC use (N)  Sweet only flavored EC use (N)  Both menthol/mint and sweet flavored EC use (N)  Menthol/mint only flavored EC use (NS) |
| Yang et al., 2022^56^ | Concurrent cigarette smoking in past 6 months (N) |
| Sharma et al., 2022^55^ | Transgender or gender diverse (N)  Ethnicity- non-Hispanic (N)  Education- some college but no degree (N)  Alcohol drinking (N)  Participatory dialogue/preparedness for quitting (P)  Self-confidence in quitting (P)  Changing physical environment/ preparedness for quitting (P)  Peer vaping (N)  (Age, race, location, income, cigarette smoking, family vaping) NS |
| Pokhrel et al., 2023^54^ | EC dependence (N)  Social network peers use EC (N)  Duration of EC use (N)  Low harm perception (N)  Preferred sensations- buzz / cognitive enhancement (N)  Preferred sensations- flavor smell and taste/ cognitive enhancement (N)  Preferred sensations- throat hit/ cognitive enhancement (N)  Preferred flavor- sweet/menthol (N)  Preferred device- open pods (N)  Nicotine concentration (N)  (Sensation seeking, past 30-day EC use, age, sex, ethnicity, working hours, anxiety, depression, past 30-day cannabis use, past 2-week binge drinking) NS |
| Lindpere et al., 2023^53^ | Intention to quit:  Reasons for use- peer/family use (P)  Reasons for use- product characteristics (N)  (Reasons for use- curiosity, replace cigarettes) NS  Past year quit attempts:  Reasons for use- curiosity (P)  (Reasons for use- peer/family use, product characteristic, replace cigarettes) NS |
| Huang et al., 2023^52^ | Increasing age (>18 years) (N) but 15-18 years (P)  Sex- Male (N)  Race- White (N)  Parental education- higher education (N)  Household restriction on smoking/ parental monitoring (P)  Past year alcohol use (N)  Past year cannabis use (N)  Past year illicit drug use (N)  Past year internalizing problems (N)  Social media follower (N) |

Abbreviation: P, positive association; N, negative association; NS, not significant; EC, e-cigarette.

***Appendix 4. Directions of the associations of predictors of intention to quit, quit attempts, and vaping abstinence.***

|  | **Intention to quit** | | | | **Quit attempts** | | | **Vaping abstinence** | | |
| --- | --- | --- | --- | --- | --- | --- | --- | --- | --- | --- |
| **Predictors** | **No. of studies with significant association/no.**  **of studies which evaluated**  **the factor** | **Overall direction of influence (no. of studies with direction of association)** | **Group of the factor** | | **No. of studies with significant association/no.**  **of studies which evaluated**  **the factor** | **Overall direction of influence (no. of studies with direction of association)** | **Group of the factor** | **No. of studies with significant association/no.**  **of studies which evaluated**  **the factor** | **Overall direction of influence (no. of studies with direction of association)** | **Group of the factor** |
| Increased age/older age | 2/11 | IC (1+, 1-) | | Probably unrelated | 0/8 | NA | Probably unrelated | 4/5 | N (1+, 3-) | Probable |
| Male sex | 2/9 | P (1+) | | Probably unrelated | 4/9 | IC (2+, 2-) | Inconsistent direction | 1/4 | N (1-) | Probably unrelated |
| Sexual orientation- heterosexual | 1/2 | P (1+) | | Possible | 1/3 | P (1+) | Possible |  |  |  |
| Gender identity- transgender or gender diverse | 1/1 | N (1-) | | Possible |  |  |  |  |  |  |
| Race -White | 3/10 | N (3-)* | | Probably unrelated | 4/7 | N (4-) | Probable | 1/2 | N (1-) | Possible |
| Ethnicity-Hispanic | 2/9 | IC (1+, 1-)* | | Probably unrelated | 4/6 | IC (2+, 1-, 1 IC) | Inconsistent direction | 0/2 | NA | Probably unrelated |
| Education | 1/2 | P (1+) | | Possible |  |  |  |  |  |  |
| Income | 0/3 | NA | | Probably unrelated | 0/2 | NA | Probably unrelated |  |  |  |
| Working hours |  |  | |  |  |  |  | 0/1 | NA | Insufficient evidence |
| Urbanicity |  |  | |  |  |  |  | 0/1 | NA | Insufficient evidence |
| Married/living with partners | 1/1 | N (1-) | | Possible | 0/1 | NA | Insufficient evidence |  |  |  |
| Migrant | 0/1 | NA | | Insufficient evidence | 1/1 | P (1+) | Possible |  |  |  |
| Parental education |  |  | |  | 0/1 | NA | Insufficient evidence | 1/1 | N (1-) | Possible |
| Location** | 0/2 | NA | | Probably unrelated |  |  |  | 1/1 | ** | Possible |
| COVID-19 outbreak | 1/1 | P (1+) | | Possible |  |  |  |  |  |  |
| Medical vs non-medical education | 0/1 | NA | | Insufficient evidence | 0/1 | NA | Insufficient evidence |  |  |  |
| Age of vaping initiation |  |  | |  |  |  |  | 0/1 | NA | Insufficient evidence |
| Duration of vaping (years) | 0/1 | NA | | Insufficient evidence | 1/1 | N (1-) | Possible | 1/1 | N (1-) | Possible |
| Level of nicotine dependence | 2/6 | IC (1+, 1-) | | Probably unrelated | 4/7 | N (3+, 4-) | Probable | 2/2 | N (2-) | Probable |
| Cravings |  |  | |  | 1/1 | P (1+) | Possible |  |  |  |
| Perceived addictiveness to vaping | 0/1 | NA | | Insufficient evidence |  |  |  |  |  |  |
| Past 30- day use of EC | 0/1 | NA | | Insufficient evidence |  |  |  | 0/1 | NA | Insufficient evidence |
| Reasons for EC use- quit smoking or other tobacco | 0/2 | NA | | Probably unrelated | 0/2 | NA | Probably unrelated |  |  |  |
| Reasons for EC use- Low cost |  |  | |  |  |  |  | 1/1 | N (1-) | Possible |
| Reasons for EC use- flavour |  |  | |  |  |  |  | 0/1 | NA | Insufficient evidence |
| Reasons for EC use- curiosity | 0/2 | NA | | Probably unrelated | 2/2 | P (2+) | Probable |  |  |  |
| Reasons for EC use- discrete | 1/1 | N (1-) | | Possible | 1/1 | N (1-) | Possible |  |  |  |
| Reasons for EC use- peer/ family vaping | 2/2 | P (2+) | | Probable | 0/2 | NA | Probably unrelated |  |  |  |
| Reasons for EC use- OTPs use | 0/1 | NA | | Insufficient evidence | 0/1 | NA | Insufficient evidence |  |  |  |
| Reasons for EC use- product characteristics | 1/1 | N (1-) | | Possible | 0/1 | NA | Insufficient evidence |  |  |  |
| Affective enhancement from EC use |  |  | |  | 0/1 | NA | Insufficient evidence |  |  |  |
| Cognitive enhancement from EC use |  |  | |  | 0/2 | NA | Probably unrelated | 1/1 | N (1-) | Possible |
| Past quit attempts |  |  | |  | 1/1 | N (1-) | Possible |  |  |  |
| EC device- disposable vs others | 2/2 | P (2+) | | Probable | 2/3 | IC (1+, 1-) | Inconsistent direction |  |  |  |
| EC device- open pods |  |  | |  |  |  |  | 1/1 | N (1-) | Possible |
| EC device- unknown | 0/1 | NA | | Insufficient evidence | 1/1 | P (1+) | Possible |  |  |  |
| Owning JUUL device |  |  | |  |  |  |  | 1/1 | N (1-) | Possible |
| Frequency of vaping | 2/3 | N (2-) | | Probable | 2/4 | N (2-) | Probable | 1/1 | N (1-) | Possible |
| Flavoured EC use | 0/1 | NA | | Insufficient evidence | 0/3 | NA | Probably unrelated | 1/2 | N (1-) | Possible |
| Sweet flavored EC use |  |  | |  |  |  |  | 2/2 | N (2-) | Probable |
| Menthol/mint flavored EC use |  |  | |  |  |  |  | 1/2 | N (1-) | Possible |
| Unaware of flavoured EC use | 1/1 | P (1+) | | Possible | 0/1 | NA | Insufficient evidence |  |  |  |
| Nicotine vs non-nicotine vaping |  |  | |  |  |  |  | 1/1 | N (1-) | Possible |
| High nicotine concentration | 1/1 | P (1+) | | Possible | 1/1 | P (1+) | Possible | 1/1 | N (1-) | Possible |
| Self-efficacy/confidence in quitting | 2/2 | IC (1+, 1-) | | Inconsistent direction | 1/1 | P (1+) | Possible |  |  |  |
| Preparedness for quitting | 1/1 | P (1+) | | Possible |  |  |  |  |  |  |
| Aware of nicotine content | 0/1 | NA | | Insufficient evidence |  |  |  |  |  |  |
| Harm perception of vaping | 6/6 | P (6+) | | Probable | 4/4 | P (3+, 1-) | Probable | 2/3 | P (2+) | Probable |
| Perceived benefits of cessation | 1/1 | P (1+) | | Possible | 0/1 | NA | Insufficient evidence |  |  |  |
| Perceived barriers of cessation | 0/1 | NA | | Insufficient evidence | 0/1 | NA | Insufficient evidence |  |  |  |
| Perceived COVID-19 threat of EC use | 1/1 | P (1+) | | Possible | 1/1 | P (1+) | Possible |  |  |  |
| Perceived COVID-19 related benefits of quitting | 1/1 | P (1+) | | Possible | 1/1 | P (1+) | Possible |  |  |  |
| Positive expectations from vaping |  |  | |  | 0/1 | NA | Insufficient evidence |  |  |  |
| Anxiety | 1/1 | N (1-) | | Possible |  |  |  | 0/1 | NA | Insufficient evidence |
| Depression |  |  | |  |  |  |  | 0/1 | NA | Insufficient evidence |
| Neuroticism | 1/1 | N (1-) | | Possible |  |  |  |  |  |  |
| Sensation seeking |  |  | |  |  |  |  | 0/1 | NA | Insufficient evidence |
| Cognitive control | 1/1 | N (1-) | | Possible | 0/1 | NA | Insufficient evidence |  |  |  |
| Internalized problems |  |  | |  |  |  |  | 1/1 | N (1-) | Possible |
| Breathing problem | 0/1 | NA | | Insufficient evidence | 0/1 | NA | Insufficient evidence |  |  |  |
| Fatigue | 1/1 | N (1-) | | Possible |  |  |  |  |  |  |
| Current cigarette smoking | 2/4 | N (2-) | | Probable | 0/4 | NA | Probably unrelated | 1/2 | N (1-) | Possible |
| Current alcohol use | 2/2 | N (2-) | | Probable |  |  |  | 1/2 | N (1-) | Possible |
| Current binge drinking |  |  | |  |  |  |  | 0/1 | NA | Insufficient evidence |
| Current cannabis use | 1/2 | N (1-) | | Possible | 1/1 | N (1-) | Possible | 1/1 | N (1-) | Possible |
| Current illicit drug use |  |  | |  |  |  |  | 1/1 | N (1-) | Possible |
| Current OTPs use | 2/3 | N (1-, 1 IC) | | Probable | 3/3 | P (2+,1-) | Probable | 2/2 | N (2-) | Probable |
| Dual use | 3/3 | N (3-) | | Probable | 3/3 | N (3-) | Probable |  |  |  |
| Poly tobacco use | 2/2 | N (2-) | | Probable | 2/2 | N (2-) | Probable |  |  |  |
| Family/household vaping | 0/1 | NA | | Insufficient evidence |  |  |  | 1/1 | N (1-) | Possible |
| Household tobacco use | 0/2 | NA | | Probably unrelated | 0/2 | NA | Probably unrelated |  |  |  |
| Parental monitoring | 1/1 | P (1+) | | Possible |  |  |  | 1/1 | P (1+) | Possible |
| Parental support | 1/1 | P (1+) | | Possible |  |  |  |  |  |  |
| Peer vaping | 2/3 | N (2-) | | Probable | 1/1 | P (1+) | Possible | 1/1 | N (1-) | Possible |
| Seeing important people vaping | 1/1 | N (1-) | | Possible |  |  |  | 1/1 | N (1-) | Possible |
| Social acceptability of vaping | 1/1 | N (1-) | | Possible |  |  |  | 0/1 | NA | Insufficient evidence |
| Overall EC marketing exposure | 0/4 | NA | | Probably unrelated | 0/2 | NA | Probably unrelated | 0/1 | NA | Insufficient evidence |
| EC accessibility | 1/1 | N (1-) | | Possible |  |  |  | 0/1 | NA | Insufficient evidence |
| Exposure to anti-vaping contents | 2/3 | P (2+, 1 IC) | | Probable | 0/1 | NA | Insufficient evidence | 0/2 | NA | Probably unrelated |
| Exposure to pro-vaping contents | 0/1 | NA | | Insufficient evidence |  |  |  |  |  |  |
| Exposure to mixed information contents | 0/1 | NA | | Insufficient evidence |  |  |  |  |  |  |
| Exposure to news content- unknown | 0/1 | NA | | Insufficient evidence |  |  |  |  |  |  |
| Social media follower |  |  | |  |  |  |  | 1/1 | N (1-) | Possible |
| Health warnings in EC package | 1/1 | P (1+) | | Possible |  |  |  | 0/1 | NA | Insufficient evidence |
| EC price affordability |  |  | |  |  |  |  | 1/1 | N (1-) | Possible |
| Getting free EC sample/coupons/exemplars | 0/1 | NA | | Insufficient evidence |  |  |  |  |  |  |
| Anxiety x fatigue | 0/1 | NA | | Insufficient evidence |  |  |  |  |  |  |
| Cravings x female sex |  |  | |  | 1/1 | N (1-) | Possible |  |  |  |
| Level of nicotine dependence x sex |  |  | |  | 0/1 | NA | Insufficient evidence |  |  |  |
| Affective enhancement from EC use x sex |  |  | |  | 0/1 | NA | Insufficient evidence |  |  |  |
| Increased age x homeless | 1/1 | P (1+) | | Possible |  |  |  |  |  |  |
| Withdrawal symptoms x homeless | 1/1 | N (1-) | | Possible |  |  |  |  |  |  |
| Sex x homeless | 0/1 | NA | | Insufficient evidence |  |  |  |  |  |  |
| Sexual orientation x homeless | 0/1 | NA | | Insufficient evidence |  |  |  |  |  |  |
| Race x homeless | 0/1 | NA | | Insufficient evidence |  |  |  |  |  |  |
| Ethnicity x homeless | 0/1 | NA | | Insufficient evidence |  |  |  |  |  |  |
| Homeless x sleeping outside | 0/1 | NA | | Insufficient evidence |  |  |  |  |  |  |
| Homeless x frequency of use | 1/1 | N (1-) | | Possible |  |  |  |  |  |  |
| Homeless x seeing other vaping | 0/1 | NA | | Insufficient evidence |  |  |  |  |  |  |
| Homeless x stress | 0/1 | NA | | Insufficient evidence |  |  |  |  |  |  |
| Homeless x harm perception | 1/1 | P (1+) | | Possible |  |  |  |  |  |  |
| Homeless x reasons for use-quit smoking | 0/1 | NA | | Insufficient evidence |  |  |  |  |  |  |
| Homeless x reasons for use-discrete | 0/1 | NA | | Insufficient evidence |  |  |  |  |  |  |
| Homeless x reasons for use-socializing | 1/1 | N (1-) | | Possible |  |  |  |  |  |  |
| Homeless x reasons for use-flavour | 0/1 | NA | | Insufficient evidence |  |  |  |  |  |  |
| Homeless x reasons for use-good psychoactive effect | 0/1 | NA | | Insufficient evidence |  |  |  |  |  |  |
| Homeless x reasons for use-nicotine content | 0/1 | NA | | Insufficient evidence |  |  |  |  |  |  |

*Some of the articles found negative association with non-Hispanic White.

**Location unspecified, because different study had different locations

***School type- Gymnasium (in Germany)

Abbreviation: P, Positive; N, Negative; NA, Not applicable; IC, Inconsistent; EC, Electronic cigarette; TM, tobacco and mint/menthol; NTM, Non-tobacco and non-mint/menthol; OTPs, Other tobacco products; ACEs, Adverse childhood experiences; ADHD, Attention deficit hyperactivity disorder; QOL, Quality of life; BMI, Body mass index.

#Intention to quit included thoughts about quit, and readiness to qui, and ranged from intention to quit in next 30 days to next 6 months.

***Appendix 5.*** ***‘Insufficient evidence’, ‘probably unrelated’ and ‘inconsistent direction’ predictors of intention to quit, quit attempts and vaping cessation.***

| **Predictors** | **Intention to quit** | **Quit attempts** | **Vaping abstinence** |
| --- | --- | --- | --- |
| Insufficient evidence | Migrant,  Medical vs non-medical education,  Duration of vaping (years),  Perceived addictiveness to vaping,  Past 30- day use of EC,  Reasons for EC use- OTPs use,  EC device type-unknown,  Flavoured EC use,  Aware of nicotine content,  Perceived barriers of cessation,  Breathing problem,  Family/household vaping,  Exposure to pro-vaping content,  Exposure to mixed information content,  Exposure to news content- unknown,  Getting free EC sample/coupons/exemplars,  Anxiety x fatigue,  Sex x homeless,  Sexual orientation x homeless,  Race x homeless,  Ethnicity x homeless,  Homeless x sleeping outside,  Homeless x seeing other vaping,  Homeless x stress,  Homeless x reasons for use-quit smoking,  Homeless x reasons for use-discrete,  Homeless x reasons for use-flavour,  Homeless x reasons for use-good psychoactive effect,  Homeless x reasons for use-nicotine content. | Married/living with partners,  Medical vs non-medical education,  Parental education,  Reasons for EC use- product characteristics,  Reasons for EC use- OTPs use,  Affective enhancement from EC use,  Unaware of flavoured EC use,  Perceived benefits of cessation,  Perceived barriers of cessation,  Positive expectations from vaping,  Cognitive control,  Breathing problem,  Exposure to anti-vaping contents,  Level of nicotine dependence x sex,  Affective enhancement from EC use x sex. | Working hours,  Urbanicity,  Age of vaping initiation,  Past 30-day use of EC,  Reasons for EC use- flavour,  Anxiety,  Depression,  Sensation seeking,  Current binge drinking,  Social acceptability of vaping,  Overall EC marketing exposure,  EC accessibility,  Health warnings in EC package. |
| Probably unrelated | Age,  Sex,  Race,  Ethnicity,  Income,  Location,  Level of nicotine dependence,  Reasons for EC use- quitting smoking or other tobacco,  Reasons for EC use- curiosity,  Household tobacco use,  Overall EC marketing exposure. | Age,  Sex,  Income,  Reasons for EC use- quitting smoking or other tobacco,  Reasons for EC use- peer/ family vaping,  Cognitive enhancement from EC use,  Flavoured EC use,  Cigarette smoking,  Household tobacco use,  Overall EC marketing exposure. | Sex,  Ethnicity,  Exposure to anti-vaping contents. |
| Inconsistent direction | Self-efficacy/confidence in quitting. | Sex,  Ethnicity,  EC device type- disposable vs others,  Current OTPs use. | Location*. |

*Location unspecified because different study had different locations.

Abbreviation: EC, E-cigarette; OTPs, Other tobacco products.

***Appendix 6. Summary of predictors of intention to quit vaping, quit attempts, and vaping abstinence by different age groups.***

| **Age group*** | **No. of studies** | **Studies** | **Predictors** | | | | |
| --- | --- | --- | --- | --- | --- | --- | --- |
|  |  |  | **Probable** | **Possible** | **Insufficient evidence** | **Probably unrelated** | **Inconsistent direction** |
| **Intention to quit** | | | | | | | |
| ~10-19 years | 6 | Ahuja et al., 2022,^39^ Dai, 2021,^58^ Wackowski et al., 2019,^37^ Zhang et al., 2020,^49^ Case et al., 2018,^51^ Lindpere et al., 2023^53^ | Reasons for EC use- peer/ family vaping,  Harm perception of vaping,  Exposure to anti-vaping contents,  Dual use,  Poly tobacco use. | Male sex,  EC device- disposable vs others,  Unaware of flavoured EC use,  Parental monitoring,  Parental support,  Health warnings in EC package,  Reasons for EC use- discrete,  Reasons for EC use- product characteristics,  Cognitive control,  Peer vaping,  Seeing important people vaping,  Social acceptability of vaping,  EC accessibility. | Sexual orientation,  Migrant,  Location**,  Duration of vaping (years),  Perceived addictiveness to vaping,  Past 30-day use of EC,  Reasons for EC use- OTPs use,  Frequency of vaping,  Flavoured EC use,  Aware of nicotine content,  Exposure to pro-vaping contents,  Exposure to mixed information contents,  Exposure to news content- unknown,  Getting free EC sample/coupons/exemplars. | Age,  Race,  Ethnicity,  Level of nicotine dependence,  Reasons for EC use- quit smoking or other tobacco,  Reasons for EC use- curiosity,  Household tobacco use,  Overall EC marketing exposure. | - |
| ~13-25 years | 7 | Alalwan et al., 2022,^50^ Jun et al., 2022,^41^ Jankowski et al., 2019,^48^ Kreslake et al., 2022,^36^  Manning et al., 2021,^35^ Tucker et al., 2020,^38^ Sharma et al., 2022^55^ | Ethnicity-Hispanic,  Harm perception of vaping,  Current cigarette smoking,  Current alcohol drinking. | Higher education,  COVID-19 outbreak,  Preparedness for quitting,  Perceived benefits of cessation,  Perceived COVID-19 threat of EC use,  Perceived COVID-19 related benefits of quitting,  Increased age x homeless,  Homeless x harm perception,  Gender identity- transgender or gender diverse,  Anxiety,  Neuroticism,  Fatigue,  Peer vaping,  Withdrawal symptoms x homeless,  Homeless x frequency of use,  Homeless x reasons for use-socializing. | Location**,  Medical vs non-medical education,  Level of nicotine dependence,  Perceived barriers of cessation,  Breathing problem,  Current cannabis use,  Family/household vaping,  Anxiety x fatigue,  Sex x homeless,  Sexual orientation x homeless,  Race x homeless,  Ethnicity x homeless,  Homeless x sleeping outside,  Homeless x seeing other vaping,  Homeless x stress,  Homeless x reasons for use-quit smoking,  Homeless x reasons for use-discrete,  Homeless x reasons for use-flavour,  Homeless x reasons for use-good psychoactive effect,  Homeless x reasons for use-nicotine content. | Sex,  Income. | Age,  Race,  Self-efficacy/confidence in quitting,  Current OTPs use. |
| ~15-35 years | 2 | Berg et al., 2021,^46^ Cuccia et al., 2021^45^ | Frequency of vaping. | Sexual orientation- heterosexual,  Level of nicotine dependence,  High nicotine concentration,  Harm perception of vaping,  Race -White,  Married/living with partners,  EC device- open pods,  Current cannabis use. | Education,  Current cigarette smoking,  Current OTPs use,  Peer vaping. | Age,  Sex,  Ethnicity. | - |
| **Quit attempts** | | | | | | | |
| ~10-19 years | 7 | Romm et al., 2022,^42^ Dai, 2021,^58^ Jankowski et al., 2019,^48^ Zhang et al., 2020,^49^ Pulvers et al., 2021,^47^ Case et al., 2018,^51^ Lindpere et al., 2023^53^ | Reasons for EC use- curiosity,  Harm perception of vaping,  Male sex,  Level of nicotine dependence,  Dual use,  Poly tobacco use. | Ethnicity-Hispanic,  Migrant,  EC device- disposable vs others,  EC device- unknown,  Race -White,  Duration of vaping (years),  Reasons for EC use- discrete,  Past quit attempts,  Current OTPs use. | Income,  Parental education,  Medical vs non-medical education,  Reasons for EC use- OTPs use,  Reasons for EC use- product characteristics,  Frequency of vaping,  Flavoured EC use,  Awareness of flavoured EC use,  Cognitive control,  Breathing problem,  Current cigarette smoking,  Exposure to anti-vaping contents. | Age,  Sexual orientation,  Reasons for EC use- quit smoking or other tobacco,  Reasons for EC use- peer/ family vaping,  Household tobacco use,  Overall EC marketing exposure. | - |
| ~18-25 years | 2 | Jun et al., 2022,^41^ Chesaniuk et al., 2019,^43^ | - | Cravings,  Self-efficacy/confidence in quitting,  Perceived COVID-19 threat of EC use,  Perceived COVID-19 related benefits of quitting,  Current OTPs use,  Cravings x female sex,  Race -White,  Harm perception of vaping | Sex,  Affective enhancement from EC use,  Cognitive enhancement from EC use,  Frequency of vaping,  Flavoured EC use,  Perceived benefits of cessation,  Perceived barriers of cessation,  Level of nicotine dependence x sex,  Affective enhancement from EC use x sex. | Current cigarette smoking. | Level of nicotine dependence. |
| ~15-35 years | 2 | Berg et al., 2021,^46^ Cuccia et al., 2021^45^ | Race -White,  Frequency of vaping. | Male sex,  Sexual orientation- heterosexual,  Level of nicotine dependence,  High nicotine concentration,  Current OTPs use,  Peer vaping,  Harm perception of vaping,  EC device- open pods,  Current cannabis use. | Income,  Married/living with partners,  Current cigarette smoking. | Age. | Ethnicity. |
| **Vaping abstinence** | | | | | | | |
| ~10-19 years | 4 | Ahuja et al., 2022 (a),^40^ Saminathan et al., 2019,^44^ Yang et al., 2022,^56^ Huang et al., 2023^52^ | Older age. | Harm perception of vaping,  Parental monitoring,  Male sex,  Race -White,  Parental education,  Nicotine vs non-nicotine vaping,  Internalized problems,  Current cigarette smoking,  Current alcohol use,  Current cannabis use,  Current illicit drug use,  Current OTPs use,  Family/household vaping,  Seeing important people vaping,  Social media follower,  EC price affordability,  Location**. | Ethnicity,  Urbanicity,  Age of vaping initiation,  Reasons for EC use- flavour,  Social acceptability of vaping,  Overall EC marketing exposure,  Health warnings in EC package,  Getting free EC sample/coupons/exemplars. | Exposure to anti-vaping contents. | - |
| ~18-29 years | 3 | Alalwan et al., 2022,^50^ Steeger et al., 2022,^57^ Pokhrel et al., 2023^54^ | Level of nicotine dependence,  Sweet flavored EC use | Harm perception of vaping,  Older age,  Duration of vaping (years),  Cognitive enhancement from EC use,  EC device- open pods,  Owning EC device,  Flavoured EC use,  High nicotine concentration,  Current OTPs use,  Peer vaping. | Sex,  Ethnicity,  Working hours,  Past 30-day use of EC,  Menthol/mint flavored EC use,  Anxiety,  Depression,  Sensation seeking,  Current binge drinking,  Current cannabis use. | - | - |

* Age groups had overlaps with each other.

**Location unspecified because different study had different locations.

Note: Red colored texts indicate positive association with outcome, blue colored texts indicate negative association with outcome.

Abbreviation: COVID-19, Coronavirus Disease 2019; EC, E-cigarette; OTPs, Other tobacco products.

**Appendix 7. Distribution of predictors by cross-sectional and longitudinal studies.**

| **Outcome** | **Predictor categories** | **Cross-sectional studies** | **Longitudinal studies** |
| --- | --- | --- | --- |
| **Intention to quit** | Probable | Disposable EC device use,  Reasons for EC use- peer/family vaping,  High harm perception of vaping,  Exposure to anti-vaping contents,  Frequency of EC use,  Current cigarette smoking,  Current alcohol drinking,  Current dual use,  Current poly tobacco use,  Peer vaping. | - |
|  | Possible | Sexual orientation-heterosexual,  Higher education,  COVID-19 outbreak,  No anxiety, no fatigue,  No neuroticism,  Less cognitive control,  Reasons for use- peer vaping,  Unaware of flavour in EC,  High nicotine concentration,  Preparedness for quitting,  Perceived benefits of cessation,  Perceived COVID-19 threat of EC,  Perceived COVID-19 related benefits of quitting,  Parental monitoring and support,  Increased age x homeless,  Harm perception x homeless,  Gender identity- transgender or gender diverse,  Married/living with partners,  Reasons for use-discrete,  Reasons for use-product features,  Anxiety,  Neuroticism,  Fatigue,  Current cannabis use,  Seeing important people vaping,  Social acceptability of vaping,  EC accessibility,  Health warnings in EC package,  Withdrawal symptoms x homeless,  Reasons for use-socializing x homeless. | Older age,  Current OTPs use. |
|  | Insufficient evidence | Same as Appendix 5. | - |
|  | Probably unrelated | Same as Appendix 5. | - |
|  | Inconsistent direction | Current OTPs use,  Self-efficacy/confidence in quitting. | - |
| **Quit attempts** | Probable | Same as Table 3. | - |
|  | Possible | Sexual orientation-heterosexual,  Migrant,  High cravings,  High nicotine concentration,  EC device-unknown,  Self-efficacy/confidence in quitting,  Perceived COVID-19 threat of EC use,  Perceived COVID-19 related benefits of quitting,  Peer vaping,  Duration of vaping (years),  Reasons for use-discrete,  Past quit attempts,  Current cannabis use,  Cravings x female sex. | Ethnicity-Hispanic,  Race-White. |
|  | Insufficient evidence | Married/living with partners,  Medical vs non-medical education,  Reasons for EC use- product characteristics,  Reasons for EC use- OTPs use,  Affective enhancement from EC use,  Unaware of flavoured EC use,  Perceived benefits of cessation,  Perceived barriers of cessation,  Positive expectations from vaping,  Cognitive control,  Breathing problem,  Exposure to anti-vaping contents,  Level of nicotine dependence x sex,  Affective enhancement from EC use x sex. | Age,  Sex,  Parental education. |
|  | Probably unrelated | Same as Appendix 5. | - |
|  | Inconsistent direction | Same as Appendix 5. | - |
| **Vaping abstinence** | Probable | - | High harm perception of vaping,  Older age,  Sweet-flavored EC use,  Level of nicotine dependence,  Current OTPs use. |
|  | Possible | Older age,  Nicotine vaping,  EC price affordability. | Parental monitoring,  Race-White,  Parental higher education,  Duration of vaping (years),  Level of nicotine dependence,  Frequency of vaping,  Reasons for EC use- low cost,  Cognitive enhancement from EC use,  EC device- open pods,  Owning JUUL device,  Flavoured EC use,  Menthol/mint flavored EC use,  Nicotine content,  Internalized problems,  Current cigarette use, OTPs use  Current alcohol use,  Current cannabis use,  Current illicit drug use,  Family/household and peer vaping,  Seeing important people vaping,  Social media follower. |
|  | Insufficient evidence | Sex,  Urbanicity,  Age of vaping initiation,  Flavoured EC use,  Harm perception of vaping,  Current cigarette use,  Exposure to anti-vaping contents. | Working hours,  Past 30-day use of EC,  Reasons for EC use- flavour,  Anxiety,  Depression,  Sensation seeking,  Current binge drinking,  Social acceptability of vaping,  Overall EC marketing exposure,  EC accessibility,  Health warnings in EC package,  Exposure to anti-vaping contents. |
|  | Probably unrelated | - | Sex,  Ethnicity. |
|  | Inconsistent direction | - | Location*. |

*Location unspecified because different study had different locations.

Note: Red colored texts indicate positive association with outcome, blue colored texts indicate negative association with outcome.

Abbreviation: COVID-19, Coronavirus Disease 2019; EC, E-cigarette; OTPs, Other tobacco products.
